# Accuracy of the discharge destination field in administrative data for identifying transfer to a long-term acute care hospital

**DOI:** 10.1186/1756-0500-3-205

**Published:** 2010-07-21

**Authors:** Jeremy M Kahn, Theodore J Iwashyna

**Affiliations:** 1 Center for Clinical Epidemiology & Biostatistics, University of Pennsylvania School of Medicine, Blockley Hall 723, 423 Guardian Drive, Philadelphia, PA 19104; 2 Division of Pulmonary & Critical Care, University of Michigan, 3A23 300 NIB, SPC 5419, 300 North Ingalls, Ann Arbor, MI 48109

## Abstract

**Background:**

Long-term acute care hospitals (LTACs) provide specialized care for patients recovering from severe acute illness. In order to facilitate research into LTAC utilization and outcomes, we studied whether or not the discharge destination field in administrative data accurately identifies patients transferred to an LTAC following acute care hospitalization.

**Findings:**

We used the 2006 hospitalization claims for United States Medicare beneficiaries to examine the performance characteristics of the discharge destination field in the administrative record, compared to the reference standard of directly observing LTAC transfers in the claims. We found that the discharge destination field was highly specific (99.7%, 95 percent CI: 99.7% - 99.8%) but modestly sensitive (77.3%, 95 percent CI: 77.0% - 77.6%), with corresponding low positive predictive value (72.6%, 95 percent CI: 72.3% - 72.9%) and high negative predictive value (99.8%, 95 percent CI: 99.8% - 99.8%). Sensitivity and specificity were similar when limiting the analysis to only intensive care unit patients and mechanically ventilated patients, two groups with higher rates of LTAC utilization. Performance characteristics were slightly better when limiting the analysis to Pennsylvania, a state with relatively high LTAC penetration.

**Conclusions:**

The discharge destination field in administrative data can result in misclassification when used to identify patients transferred to long-term acute care hospitals. Directly observing transfers in the claims is the preferable method, although this approach is only feasible in identified data.

## Objective

Long-term acute care (LTAC) hospitals specialize in the care of severely ill hospitalized patients with longer than average lengths of stay [1]. Typically LTACs provide care for patients with complex care needs after an episode of severe acute illness, such as patients requiring intensive wound care or prolonged mechanical ventilation [2]. LTACs are among the fastest growing segments of the US health care system, increasing at an average rate of approximately 10% per year [3]. Despite such growth, it is not clear whether or not LTACs provide value over the alternatives sites of care such as skilled nursing facilities, rehabilitation hospitals, or intermediate care units within acute care hospitals [4]. Research is needed to examine the factors related to LTAC utilization and the outcomes of patients transferred to LTACs.

Large, multi-center administrative datasets are an important resource for research on the organization of care [5]. Yet administrative data frequently do not contain direct patient identifiers, making it impossible to identify transfers to LTACs. An alternate approach is to use the "discharge destination" field, which is commonly available in administrative data and usually contains an LTAC-specific code. However, administrative data often contain coding errors [6], and whether or not the discharge destination field accurately identifies transfer to an LTAC is unknown. Prior to using the discharge destination field to perform LTAC-related research, it is important to better understand its performance compared to more direct methods of identifying transfers. The objective of this study was to determine the accuracy of the discharge destination field in administrative data, compared to the reference standard of directly observing such transfers in the data.

## Methods

We performed a cohort study to determine the accuracy of the discharge destination field administrative data for identifying patients transferred to an LTAC after an acute care hospitalization. We used the 2006 United States Medicare Provider Analysis and Review (MedPAR) file, which contains patient-level clinical and demographic data for all hospitalizations of fee-for-service Medicare beneficiaries in the United States. MedPAR is a unique data source for this project, since it includes not only a discharge destination field specifying the location of the patient after transfer ("DSTNTNCD") but also direct patient identifiers which allow tracking of specific individuals across multiple hospitalizations, including hospitalizations in an LTAC. Thus we were able to compare LTAC transfers as defined in the discharge destination field to the reference standard to directly observing LTAC transfers in the administrative record.

All hospitalizations in an adult general medical-surgical hospital during 2006 were eligible for the analysis. We excluded patients < 65 years of age, which are not typical of the elderly Medicare population, and patients hospitalized in Alaska and Hawaii, which have limited access to LTACs because of their unique geography. We categorized the discharge destination field into six mutually exclusive categories: home, skilled nursing facility or rehabilitation hospital, another acute care hospital, an LTAC, deceased, and other or unknown. Discharge to an LTAC was based on code 63, "Discharge/transferred to a long term care hospital"), which is present in Medicare claims since 2002.

Independent from the discharge location field, we determined whether or not the patient actually was transferred to an LTAC by directly observing such transfers in the claims. For this step, LTACs were identified using hospital characteristics from the 2006 Centers for Medicare and Medicaid Health Cost Reporting Information System (provider type = general long-term) and the provider characteristics embedded in the MedPAR hospital provider number (provider type = general long-term). These data sources can both be used to identify long-term acute hospitals. For hospitals in which the two data sources did not agree (27 of 6,680, 0.4%), we performed internet searches and placed telephone calls to confirm the hospital type. We defined LTAC transfers as temporally adjacent hospitals (i.e. discharge from the first hospital on date n and admission to the second hospital on date n or n +1), in which the first hospitalization is in a short stay hospital and the second hospitalization is in an LTAC [7].

We then created 2 × 2 contingency tables to determine the sensitivity, specificity, positive predictive value and negative predicted value of the discharge destination field compared to the reference standard of directly observing the LTAC transfer. We calculated exact confidence intervals for each value using the binomial distribution. We performed the analysis in three groups of patients: all acute care hospitalizations, the subset of acute care hospitalizations involving an intensive care unit (ICU) admission [8], and the subset of ICU patients receiving mechanical ventilation [9]. The last two groups were examined because LTAC utilization is particularly high in these groups, and therefore the performance characteristics of the discharge codes might vary from the general population. Finally, we repeated all analyses in Pennsylvania, a US state with relatively high LTAC penetration. All analyses were performed in Stata 11.0 (College Station, Texas, US). The University of Pennsylvania Institutional Review Board approved this research.

## Results

Table 1 shows a tabulation of the discharge destination field in MedPAR categorized by whether or not the patient was actually transferred to an LTAC as observed in the claims. Nationwide 0.8% of acute care hospitalizations ended in a transfer to an LTAC. A higher proportion of hospitalizations involving intensive care (2.3%) and mechanical ventilation (8.3%) ended in an LTAC transfer. Slightly higher transfer rates were observed in Pennsylvania. In general, LTAC transfers misclassified by the discharge destination field (i.e. the false negatives) were identified as being transferred to a skilled nursing facility, rehabilitation hospital or another acute care hospital (Table 1). For example, in the entire US sample, of 19,543 false negatives, 11,854 (60.7%) were listed as discharged to a skilled nursing facility or rehabilitation hospital and 5,870 (30.0%) were listed as discharged to another short-term hospital.

**Table 1:** Contents of the discharge destination field in Medicare categorized by actual transfer to a long-term acute care hospital.

	Transferred to LTAC	Not transferred to LTAC
**United States**	**(n = 86,105)**	**(n = 9,965,336)**
Home	1,436	6,566,321
Skilled care/rehabilitation	11,854	2,489,062
Dead	264	595,109
Short term hospital	5,870	279,631
LTAC	66,562	25,110
Unknown	119	10,103
		
**United States, ICU only**	**(n = 40,600)**	**(n = 1,699,545)**
Home	450	955,182
Skilled care/rehabilitation	5,439	397,987
Dead	55	253,580
Short term hospital	3,108	83,476
LTAC	31,528	7,652
Unknown	20	1,668
		
**United States, ventilated only**	**(n = 19,938)**	**(n = 221,188)**
Home	104	47.661
Skilled care/rehabilitation	2,582	54,270
Dead	8	105,320
Short term hospital	1,809	10,258
LTAC	15,420	3,468
Unknown	15	211
		
**Pennsylvania**	**(n = 4,458)**	**(n = 490,899)**
Home	65	313,799
Skilled care/rehabilitation	525	136,266
Dead	1	26,707
Short term hospital	338	12,259
LTAC	3,495	1,366
Unknown	34	502
		
**Pennsylvania, ICU only**	**(n = 2,107)**	**(n = 80,052)**
Home	21	41,724
Skilled care/rehabilitation	246	22,268
Dead	0	11,820
Short term hospital	149	3,745
LTAC	1,690	432
Unknown	1	63
		
**Pennsylvania, ventilated only**	**(n = 1,216)**	**(n = 10,473)**
Home	4	2,020
Skilled care/rehabilitation	140	2,909
Dead	0	4,863
Short term hospital	95	438
LTAC	976	232
Unknown	1	11

ICU = intensive care unit; LTAC = long-term acute care hospital

Compared to the reference standard of directly observing transfers in the claims, the discharge destination field was modestly sensitive but highly specific (Table 2). Across all patient categories in the United States sensitivity ranged from 77.3% to 77.7% and the specificity ranged from 98.4% to 99.7%. The positive predictive value ranged from 72.6% to 81.6%, and as expected was higher in the higher prevalence groups. Due to the relatively low prevalence, negative predictive value approached 100%. Compared to hospitalizations in the US as a whole, in Pennsylvania the sensitivity was slightly higher with similar specificity.

**Table 2:** Performance characteristics of the discharge destination field for identifying patients transferred to a long-term acute care hospital after an acute care hospitalization.

	Transfer Prevalence, %	Sensitivity, % (95% CI)	Specificity, % (95% CI)	PPV, % (95% CI)	NPV, % (95% CI)	+ LR (95% CI)	- LR (95% CI)
United States	0.8	77.3 (77.0 - 77.6)	99.7 (99.7 - 99.8)	72.6 (72.3 - 72.9)	99.8 (99.8 - 99.8)	307 (303 - 311)	0.23 (0.23 - 0.23)
United States, intensive care only	2.3	77.7 (77.3 - 78.1)	99.6 (99.5 - 99.6)	80.5 (80.1 - 80.9)	99.5 (99.5 - 99.5)	172 (169 - 176)	0.22 (0.22 - 0.23)
United States, ventilated only	8.3	77.3 (76.8 - 77.9)	98.4 (98.4 - 98.5)	81.6 (81.1 - 82.2)	98.0 (97.9 - 98.0)	49.3 (47.7 - 51.0)	0.23 (0.22 - 0.24)
Pennsylvania	0.9	78.4 (77.2 - 79.6)	99.7 (99.7 - 99.7)	71.9 (70.6 - 73.2)	99.8 (99.8 - 99.8)	28.9 (27.3 - 30.5)	0.22 (0.21 - 0.24)
Pennsylvania, intensive care only	2.6	80.2 (78.4 - 81.9)	99.5 (99.4 - 99.5)	79.6 (77.9 - 81.3)	99.5 (99.4 - 99.5)	149 (135 - 164)	0.20 (0.18 - 0.22)
Pennsylvania, ventilated only	10.4	80.3 (77.9 - 82.5)	97.8 (97.5 - 98.1)	80.8 (78.5 - 83.0)	97.7 (97.4 - 98.0)	36.2 (31.8 - 41.3)	0.20 (0.18 - 0.23)

CI = confidence interval; PPV = positive predictive value; NPV = negative predictive value; LR = likelihood ratio. All values percents.

## Discussion

We found that the discharge destination field in administrative data was only modestly accurate in identifying patients transferred to long-term acute care hospitals. The specificity of the test was high, resulting in a relatively low false positive rate and high negative predictive value. However, the sensitivity was somewhat low, resulting in a high false negative rate and low positive predictive value. When false negatives occurred, the patients were most frequently classified as having been transferred to skilled nursing facilities, inpatient rehabilitation hospitals or acute care hospitals rather than LTACs. The performance characteristics of the discharge destination field were consistent across key subgroups of patients, indicating that coding error was not conditional on prevalence of LTAC utilization.

These results have important implications for LTAC-related research. Ideally, investigators using administrative data to study LTACs should only use data with direct patient identifiers that allow tracking of patients across hospitalizations. Unfortunately, due to privacy restrictions and other data constraints, few administrative hospital discharge data sets contain this information [5]. For example, US state discharge data sets like those available in the Agency for Healthcare Research and Quality's Healthcare Costs and Utilization Project do not have this capability. Researchers that must use unidentified hospitalization data to study LTACs should recognize the limitations of the discharge destination field for identifying LTAC transfers. Sensitivity analyses that account for false negatives and other classification errors are necessary to understand how such errors could potentially bias results. For investigations in which accurate identification of LTAC transfer is crucial, the limitations the discharge destination field in unidentified administrative data may preclude its use.

For research that uses the discharge location field to identify LTACs, the implications of misclassification will depend on how researchers use the field. Given the high positive predictive value, researchers that use LTAC transfer as an outcome (i.e. patient factors associated with transfer to an LTAC) can be reasonably certain that patients meeting the outcome are true positives. Assuming non-differential misclassification, the misclassification serves mainly to decrease power. However, if a researcher wishes to study the incidence or outcomes of patients transferred to LTACs, the high false negative rate would mean that a substantial number of patients would be missed. Researchers should exercise particular caution in this instance. In either case, the degree to which misclassification is differential (i.e. systematically conditional on hospital or patient level factors) will lead to potentially important bias. Future studies should examine whether misclassified patients differ in fundamental ways from correctly classified patients.

Our study has several limitations. We analyzed only one administrative data source. The performance characteristics of the discharge destination field may differ among different data sources. Nonetheless, given the historical importance of Medicare data for hospital reimbursement and health services research, we strongly doubt that they are systematically less accurate than other administrative data. We also used a potentially imperfect reference standard. Although our method should capture nearly all LTAC transfers, we could misclassify patients with incorrectly coded admission and discharge dates, or patients admitted to LTACs through means other than direct transfers, an extremely rare occurrence [10]. Additionally, we could not determine the true discharge destination of false positives (i.e. patients thought to have undergone LTAC discharge by the discharge destination field but who did not actually under LTAC transfer) or determine the patient-level factors associated with misclassification. Future research that fills these knowledge gaps may help researchers understand the implications of misclassification when using the discharge destination field, perhaps expanding the role of unidentified data in LTAC research. Finally, LTACs as a hospital type are specific to the United States; our findings are not applicable to other countries.

In conclusion, the discharge destination field in administrative data can result in misclassification of patients transferred to long-term acute care hospitals. Directly observing transfers in the claims is the preferable method, although this approach is only feasible in identified data.

## Competing interests

Dr. Kahn is employed by the University of Pennsylvania, which owns and operates a long-term acute care hospital under a cooperative agreement with Good Sheppard Rehabilitation Network--both are non-profit entities. Dr. Kahn also receives grant funding from the United States National Institutes of Health to study long-term acute care hospitals. Dr. Iwashyna reports no competing financial interests.

## Authors' contributions

JK designed the study, analyzed the data, interpreted the results and drafted the manuscript. TI obtained the data, provided input into study design, interpreted the results and critically revised the manuscript for important content. All authors read and approved the final manuscript.

## Funding

Funded by R01 HL096651 from the United States National Institutes of Health (Kahn). Drs. Kahn and Iwashyna are supported by a career development awards from the United States National Institutes of Health (K23 HL096651, Kahn; K08 HL091249, Iwashyna). This study was also funded in part from a grant from the Pennsylvania Department of Health, which specifically disclaims responsibility for any analyses, interpretations or conclusions.
